# The Role of Aryl Hydrocarbon Receptor in the Endothelium: A Systematic Review

**DOI:** 10.3390/ijms241713537

**Published:** 2023-08-31

**Authors:** Sol Guerra-Ojeda, Andrea Suarez, Alicia Valls, David Verdú, Javier Pereda, Elena Ortiz-Zapater, Julián Carretero, Maria D. Mauricio, Eva Serna

**Affiliations:** 1 Department of Physiology, University of Valencia, 46010 Valencia, Spain; solanye.guerra@uv.es (S.G.-O.); ansuafor@alumni.uv.es (A.S.); avallsa3@alumni.uv.es (A.V.); david.verdu@uv.es (D.V.); javier.pereda@uv.es (J.P.); julian.carretero@uv.es (J.C.); eva.serna@uv.es (E.S.); 2 Biomedical Research Institute INCLIVA, University of Valencia, 46010 Valencia, Spain; elena.ortiz-zapater@uv.es; 3 Department of Biochemistry and Molecular Biology, University of Valencia, 46010 Valencia, Spain

**Keywords:** AhR, endothelium, aorta, endothelial function, vascular homeostasis, cardiovascular system

## Abstract

Activation of the aryl hydrocarbon receptor (AhR) has been shown to be important in physiological processes other than detoxification, including vascular homeostasis. Although AhR is highly expressed in the endothelium, its function has been poorly studied. This systematic review aims to summarise current knowledge on the AhR role in the endothelium and its cardiovascular implications. We focus on endogenous AhR agonists, such as some uremic toxins and other agonists unrelated to environmental pollutants, as well as studies using AhR knockout models. We conclude that AhR activation leads to vascular oxidative stress and endothelial dysfunction and that blocking AhR signalling could provide a new target for the treatment of vascular disorders such as cardiovascular complications in patients with chronic kidney disease or pulmonary arterial hypertension.

## 1. Introduction

### 1.1. Background

Historically, the aryl hydrocarbon receptor (AhR) is known for its function in the metabolism of xenobiotics. As we will describe, this is a very limited view of the functions of this receptor, and several studies have associated AhR activation with important pathologies such as cancer [1] or even with genetic malformations [2,3]. One of the highest affinity agonists for AhR is the molecule 2,3,7,8-tetrachlorodibenzo-para-dioxin (TCDD), which causes harmful effects on human health. However, AhR activation is not only triggered by the action of agonists from environmental chemicals but also by food and endogenous substances. In fact, in the absence of exogenous ligands, the intrinsic activity of the AhR depends on endogenous ligands present in the cell. Tryptophan derivatives, UV-derived photoproducts, tetrapyrroles such as bilirubin, some arachidonic acid metabolites such as lipoxin A4 and prostaglandin G, modified low-density LDL protein, and carotenoids are some of its endogenous ligands [4,5]. AhR endogenous agonists may vary according to cell type and stage of development, attributing different effects to the activation of the AhR pathway. For example, constitutive activation of AhR has been shown to be essential in physiological processes such as embryogenesis, neurogenesis, circadian rhythm, aging, metabolism, and response to hypoxia [6,7]. It has been implicated in the degradation of steroid hormone receptors by the proteasome, in the cellular response to UVB stress, and in the differentiation of certain T cell subtypes [8,9,10]. Moreover, its constitutive expression in most vertebrates, both aquatic and terrestrial, its presence in almost all mammalian cell types, and its high structural homology suggest that it is a phylogenetically very primordial gene. Thus, the *AhR* gene is highly conserved by evolution and has a pre-existing origin prior to the appearance of dioxins in the environment [11,12,13]. All these findings show that it is very likely that AhR has a physiological role in systems whose main function is not detoxification. This is supported by studies in AhR knockout (AhR KO) mice that exhibit cardiac hypertrophy [14,15], abnormal hepatocyte and sinusoid morphology [16], and vascular disorders [17] such as failure in the developmental closure of the ductus venosus [18].

### 1.2. Mechanism of Action of AhR

The human AhR gene encodes a protein of 848 amino acids with a molecular mass of 96 KDa. It is identical to the mouse AhR gene at the N-terminal end, and they are 60% homologous at the C-terminal end [19]. AhR forms a heterodimer with the Aryl Hydrocarbon Receptor Nuclear Translocator (ARNT), and AhR repressor (AHRR) is a negative regulator of AhR. They belong to the bHLH/PAS (basic Helix-Loop-Helix/PER-ARNT-SIM) superfamily of transcription factors.

**Figure 1:**
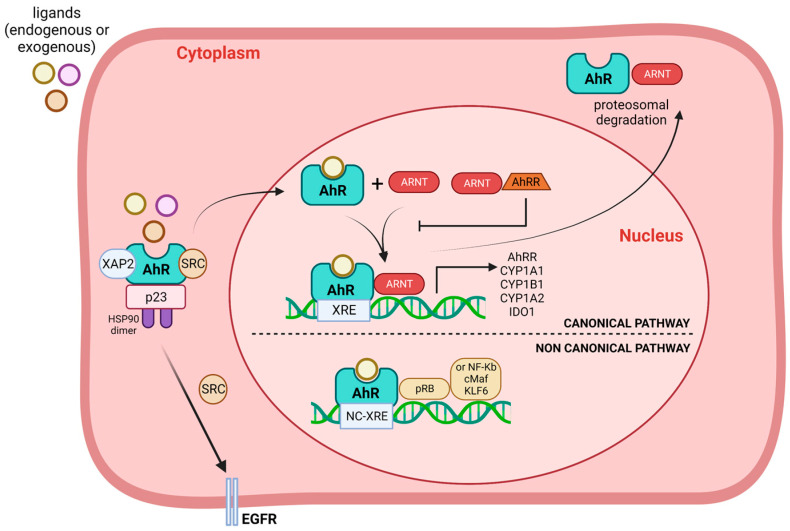
Schematic representation of the canonical and non-canonical signalling pathways of AhR. AhR is found in the cytosol in its latent form associated with a protein complex (HSP90: heat shock protein, XAP2: hepatitis B virus X-associated protein, SRC: Proto-Oncogene tyrosine-protein kinase s-Src and p23). After activation by ligands, it translocates to the nucleus and forms a heterodimer with AhR nuclear translocator (ARNT-), which acts as a transcriptional factor, increasing gene expression contained in the Xenobiotic response element (XRE). This is the canonical pathway and affects the expression of genes such as CYP1A1, CYP1B1, CYP1A2, AhR repressor (AhRR), or indoleamine 2,3-dioxygenase (IDO1). By non-canonical pathway, AhR binds other proteins such as pRB, NF-κB, c-Maf, or KLF6 and increases the expression of genes contained in the Non-consensus Xenobiotic response element (NC-XRE). Moreover, when AhR is activated by ligands, the release of c-Src (SRC) can activate epidermal growth factor receptor (EGFR) signalling. Finally, AhR is degraded by proteasome in the cytosol, which is one of the possible degradation pathways. Image created using BioRender.

The canonical signalling pathway begins in the cytosol where AhR is found in its latent form associated with a protein complex (proto-oncogene tyrosine-protein kinase (c-Src), heat shock protein 90 (HSP90), p23, and the hepatitis B virus X-associated protein 2 (XAP2) also known as aryl hydrocarbon receptor interacting protein, (AIP)) that stabilizes it and prevents its degradation by the proteasome in the absence of ligand (Figure 1). When AhR is activated by ligand binding, there is a change in the cellular compartmentalization of the receptor, which rapidly accumulates into the nucleus. Once in the nucleus, the AhR is released from binding to the protein complex and interacts with the ARNT protein to form a transcriptionally active heterodimer (AhR/ARNT), which interacts with cellular DNA through Xenobiotics or Dioxin Response Elements (XREs or DREs) sequences and increases the expression of the genes that contain them [20]. The most studied target genes are those encoding xenobiotic metabolizing enzymes, such as CYP1A1 and CYP1B1. On the other hand, the non-canonical signalling pathway involves AhR-dependent activation of epidermal growth factor receptor (EGFR) via release of c-Src, retinoblastoma protein (Rb), NF-κB, c-Maf, KLF6 among others that recognize a novel non-consensus XRE (NC-XRE), emphasizing a difference from the XRE-dependent AhR signalling mechanism (Figure 1) [21,22,23].

AhR signalling is specific to the type of cell, tissue, or predominant conditions, and it also varies depending on the ligand that activates it [24]. 

### 1.3. The Role of AhR in Cardiovascular System

According to the latest report of the World Health Organisation, cardiovascular diseases (CVDs) are the leading cause of death globally, taking an estimated 17.9 million lives each year, representing 27% of all global deaths [25]. Controlling risk factors such as hypercholesterolemia, hyperglycaemia, obesity, hypertension, or smoking reduces the number of acute vascular complications and death from CVDs. All these risk factors have endothelial dysfunction in common [26]. The endothelium is a single layer of epithelium that lines the inside of blood vessels. It is a semi-permeable barrier with intercellular junctions, mainly tight junctions, that regulates the passage of molecules across the vascular wall [27,28,29]. Among the various properties attributed to the endothelium are the maintenance of vascular tone, regulation of blood flow, maintenance of an antithrombotic state, and participation in the immune response [27]. Thus, the endothelium plays an important role in vascular homeostasis, and its dysfunction is the origin of CVDs. AhR is known to be expressed at the vascular level [30] in both endothelium and smooth muscle [31], and it seems that in the endothelium, the expression is abundant [32]. However, the role and function of the AhR in the vessel, particularly in the endothelium, have been poorly studied. Therefore, this systematic review aims to summarize current knowledge on AhR signalling in the endothelium and its cardiovascular implications. 

We will focus on endogenous AhR agonists, such as some uremic toxins, as well as other agonists unrelated to environmental pollutants. We will analyse the effect of the AhR activation pathway on endothelial function, as well as studies using AhR KO models.

## 2. Methods

This systematic review was conducted between 1 December 2022 and 31 December 2022 through experiments, comparative analyses, and observational studies that focused on the effects of several AhR agonists in the endothelium, according to PRISMA 2020 guidelines for Systematic Reviews [33]. The eligible papers published until 2022 were searched in several databases, including PubMed and Scopus.

### 2.1. Study Selection

The following inclusion criteria were predefined for screening papers by title and abstract: (1) research paper and (2) exclusive use of human, rodent, lagomorph, and porcine subjects. The papers included were written in English and described the signalling pathways of AhR in the endothelium. 

The exclusion criteria were (1) publication in languages other than English or Spanish, (2) use of exogenous agonists from environmental pollutants such as TCDD, dioxins, Benzo[a]pyrene and polychlorinated biphenyls (PCBs), (3) papers focusing on cancer and (4) inadequacy of the data presented or poor description of the methods applied. In addition, expert opinions, editorial letters, systematic reviews, meta-analyses, case reports, and qualitative studies were removed from the review process.

### 2.2. Search Strategy

All studies indexed in PubMed and Scopus from 1 January 2008 to 31 December 2022 describing the effects of several AhR agonists in the endothelium were identified. Search terms included the following: Aryl hydrocarbon receptor AND endothelium. 

First, the titles and abstracts of all papers were reviewed, and those related to the topic of interest were selected. Next, the full manuscripts of the selected papers were obtained, and those that appeared to meet the inclusion criteria were reviewed. Finally, after the exclusion of the irrelevant papers, all eligible studies were formally assessed and included in this systematic review. The related data, such as the authors and time of study, experimental model, sample, AhR agonist and antagonist, and main results, were extracted from all the included papers. Any disagreement at the title/abstract or full manuscript screening stages was resolved by the two authors independently.

## 3. Results and Discussion

### 3.1. Literature Search Results

**Figure 2:**
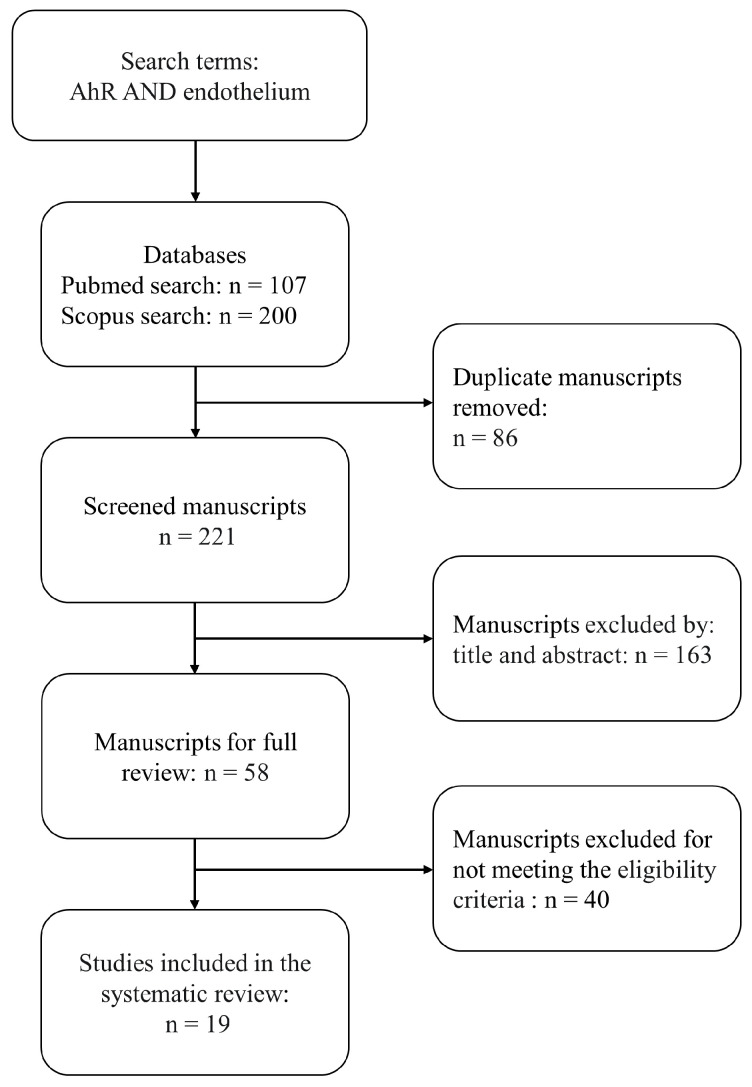
Search flow diagram for systematic review.

The searches retrieved 307 relevant papers, and after deduplication, 221 manuscripts were screened based on the title and abstract. A further 163 papers were excluded because they did not meet the inclusion criteria. Next, we evaluated the full text of 58 potentially relevant studies, of which 40 were excluded because they used exogenous agonists from environmental pollutants or focused on cancer. A total of 19 studies were included in the final systematic review and evaluated on the basis of the data extracted. The complete flow chart and checklist describing the study selection process are shown in Figure 2.

The data extracted from the included studies are summarized in Table 1. To provide insights into the role of AhR in the endothelium, the studies were grouped into sections according to AhR agonist type or knockout models.

### 3.2. Uremic Toxins Such as Indoxyl Sulfate and Indole-3-Acetic Acid as Agonists of AhR 

This section reviews studies linking endothelial function with uremic toxins such as indoxyl sulfate (IS) and indole-3-acetic acid (IAA), which are AhR agonists and have been related to cardiovascular complications associated with chronic kidney disease (CKD). 

Nakagawa et al. [47] showed IS-induced superoxide anion (-O_2_^●−^) production via AhR-NADPH oxidase pathway and a decreased vasodilation in response to both acetylcholine and sodium nitroprusside in rat thoracic aorta. They used different concentrations and incubation times of IS, concluding that IS (500 µM) incubated for 4 h was compatible with the IS blood concentration in patients with end-stage CKD. The lower vasodilation to acetylcholine induced by IS was partially reversed by the AhR antagonist (CH-223191), whereas the lower vasodilation to sodium nitroprusside in endothelium-denuded aortic rings was not affected by an AhR antagonist. The authors hypothesize that these differences could be due to the higher expression of AhR in endothelial cells (ECs) compared to smooth muscle cells. Likewise, Koizumi et al. [41] reported that IS (500 µM) induced oxidative stress by activation of NADPH oxidase 4 and decreased the intracellular nicotinamide phosphoribosyltransferase (iNampt) and Sirtuin 1 activity, resulting in cellular senescence in human umbilical vein endothelial cells (HUVECs). The authors demonstrated that IS incubation increased acetylated p53, with no changes in total p53 protein expression, which suggests that Sirtuin 1 activity is decreased. Sirtuin 1 is a NAD+-dependent deacetylase and a key enzyme delaying senescence and oxidative stress. The results from Koizumi et al. showed that IS decreased Sirtuin 1 activity and NAD+ levels. The authors demonstrated that the reduction of NAD+ levels was due to a decrease in the iNampt activity, the rate-limiting enzyme for NAD+ synthesis. It is known that oxidative stress reduces NAD+ levels, so their results indicated that IS induces oxidative stress and senescence in HUVECs. Moreover, the authors demonstrated that apocynin, a NADPH oxidase inhibitor, restored the activities of Sirtuin 1 and iNampt, and NAD content, all reduced by IS. Finally, AhR inhibition reversed these effects, concluding that AhR activation by IS promotes endothelial oxidative stress and senescence. Precisely, the enhancement of oxidative stress following IS-induced NADPH oxidase activation lead to senescence via iNampt-NAD(+)-Sirtuin 1 system. Consequently, its blockade may be useful for the treatment of carviovascular complications in patientes with CKD.

Nguyen et al. [49] also found that IS caused endothelial dysfunction by increasing oxidative stress in rat thoracic aorta at 300 µM but not at 10–100 µM. Their results showed a lower vasodilation to acetylcholine in the presence of IS (300 µM), which was reversed by the AhR antagonist (CH-223191) at 1 and 10 µM. They also found that IS increased endothelial NADPH oxidase 4, nitrotyrosine, and O_2_^●−^ expression and reduced endothelial nitric oxide synthase (eNOS) expression. All these effects were prevented in the presence of the AhR antagonist. The authors established that the mechanism implicated in the endothelial dysfunction induced by IS was the activation of the AhR-CYP1A1 pathway.

On the other hand, patients with CKD have a prothrombotic state with an increase in some procoagulant factors, including tissue factor (TF). Based on this scenario, Gondouin et al. [38] studied TF expression in HUVECs incubated with the uremic toxins IS (1 mM) and IAA (50 µM) at concentrations similar to those found in patients with CKD. Their results showed an increased expression of TF and upregulation of eight genes regulated by the AhR, an increase in TF-dependent procoagulant activity, and a greater release of microparticles with procoagulant activity from ECs. Using geldanamycin, which inhibits the ATPase activity of Hsp90 essential to protect AhR from proteolysis, IS- and IAA-induced TF expression was reduced. In contrast, by inhibiting the NF-κB pathway, IS- and IAA-induced TF expression was reduced but not completely. Their study also showed that AhR was rapidly degraded after IS and IAA treatment, whereby AhR is activated, translocates to the nucleus, and decreases its protein levels. This decrease in AhR protein levels is maintained longer with IS than with IAA. To elucidate the underlying mechanisms, they used an indirect AhR inhibitor, geldanamycin, and silenced it and found that TF production decreased, as well as procoagulant activity. They concluded that IS and IAA increase TF via AhR and that the procoagulant effect of TF and the pro-atherogenic effect of AhR may be involved in atherogenesis in CKD patients. 

CKD is also linked to vascular inflammation and impaired immune response, and uremic toxins may contribute to worsening this process. Dou et al. [35] observed increased IAA serum levels in patients with CKD (0.7–3.9 µM for control vs. 0.6–18.5 µM for CKD patients). Furthermore, they found that the incubation of IAA at 5, 10, 25, and 50 µM increased COX-2 expression in HUVECs and other cell lines such as cardiac-derived microvascular (HMVEC-C), coronary artery (HCAEC), and aortic (HAoEC) endothelial cells. The authors concluded that the pathway involved was AhR/p38 MAPK/NFKB and that elevated serum levels of IAA could be a predictor of cardiovascular mortality in patients with CKD. Moreover, the authors found that IAA increases ROS production in endothelial cells, and this effect was only moderately reversed by the AhR inhibitor, CH-223191 10 μM, and markedly reversed by the ROS scavenger N-acetylcysteine. Therefore, IAA has prooxidant effects in endothelial cells, but AhR activation seems not to be the only mechanism implicated. Ito et al. [39] suggested that IS caused vascular inflammation and increased ROS production in control AhR mice, which was further aggravated after the treatment with TNF-α. Nevertheless, they demonstrated that IS-induced AhR activation mediates endothelial inflammation and leukocyte recruitment via activator protein-1 (AP-1) to induce E-selectin since AP-1 transcriptional activity and TNF-*α*-induced E-selectin expression were increased in HUVECs but not in HUVECs transfected with AhR-specific siRNA (siAhR). The mechanism seems to be mediated by AhR since inflammation and leukocyte recruitment were decreased in a model of endothelial cell-specific AhR KO (ECAhR) mice. Similarly, Kim et al. [40] incubated monocytes from end-stage CKD patients with IS for 48 h, and then the monocyte-conditioned media was added to HUVEC for 4 h. They observed in HUVECs that IS-induced proinflammatory cytokines led to the secretion of CX3CL1, a chemokine ligand of CX3CR1 that is highly expressed on CD4 + CD28 − T cells, which are the predominant cell type in end-stage KD patients. CX3CL1 was also increased in HUVECs in the presence of TNF-α, which was related to leukocyte recruitment.

As we can see, most of the articles in this section focus on IS-induced activation of the AhR pathway during CKD; however, physiological factors such as shear stress could also activate the AhR pathway. In this regard, Lano et al. [42] designed a study comparing the activation of AhR by fluid shear stress, considered a physiological process, versus IS incubation, considered toxicological, in HUVECs. They found that laminar shear stress (5 dynes/cm^2^) increased the expression of both AhR and AhRR. In contrast, IS (200 µM) did not affect AhR expression but increased AhRR expression. Both shear stress and IS up-regulated COX2, as well as CYP1A1 and CYP1B1 mRNA expression, in an AhR-dependent manner because the effects of SS and IS on the gene expression, were reverted by silencing AhR and using Genistein, an inhibitor of AhR nuclear translocation. However, the decrease in COX2 with Genistein was not as exaggerated, suggesting that COX2 production can be through both canonical and non-canonical pathways. They found that SS and IS increased TF expression but that TF only has a procoagulant effect when activated by IS. Furthermore, IS did not induce AhR binding to the F3 promoter (gene encoding for endothelial tissue factor), supporting that the regulation of TF via IS/AhR is mediated by a non-canonical pathway. They conclude that SS and IS activate AhR by different mechanisms and that TF activation by IS has a procoagulant effect, whereas SS does not, suggesting that TF induction mediated by the IS/AHR axis is different from that mediated by the SS/AHR axis. This work shows that depending on the agonist that activates AhR signalling, the physiological effect can vary.

To summarise, all these articles show the contribution of uremic toxins, as AhR agonists, to increased oxidative stress and vascular dysfunction. Most of these studies have been done in vitro, trying to mimic a situation in CKD. We believe it is important to note the different concentrations used. In that sense, Gondouin et al. [38] or Kim et al. [40] used an IS concentration of 1000 µM when other authors reported levels of IS of around 500 µM in patients with CKD [53]. According to Dou et al. [35] IAA levels in CKD patients range between 0.6–18.5 µM, whereas Gondouin et al. [38] performed their experiments with IAA at 50 µM.

### 3.3. Non-Uremic Toxins: Effect of Other Agonists of AhR

The uremic toxins are important in the context of CKD; however, there are other molecules, both endogenous and exogenous, whose function is not related to diseases, which have shown the ability to activate this pathway. In order to elucidate their physiological role, in this section, we will review the effects of kynurenine, ITE, and I3C on the endothelium. 

Kynurenine: This molecule is an AhR agonist involved in cellular energy generation. It is synthesised from tryptophan through the action of indoleamine 2,3-dioxygenase (IDO1). Kynurenine promotes vascular development and angiogenesis by up-regulating vascular endothelial growth factor A (VEGFA), which is essential for cardiac regeneration. Failure in kynurenine metabolism has been linked to cardiovascular diseases, among others. The human neonatal heart has regenerative capacity, a function that declines rapidly after birth. Reactivation of cardiomyocyte proliferation may be an interesting strategy for repairing cardiac damage. In this regard, the study by Zhang et al. [51] proposed that activation of the kynurenine pathway may be essential for cardiomyocyte proliferation in neonatal heart regeneration. In their study, they treated mouse cardiomyocytes (CMs) with IFNγ for 24 h in order to upregulate IDO1 expression. Then, CMs were co-cultured with cardiac microvascular endothelial cells (CMECs) and observed an increase in kynurenine levels in the CMECs, suggesting that there was a kynurenine transport between both types of cells. Thus, kynurenine produced by CMs reaches the endothelium, where it binds to AhR and promotes its translocation to the nucleus, increasing VEGFA gene expression and contributing to cardiac regeneration. To further demonstrate the beneficial effects of kynurenine in promoting cardiac generation in neonates via the AhR pathway, the author used the AhR KO mouse. In this sense, VEGFA staining was reduced in cardiac endothelium from the AhR KO mice, confirming their hypothesis. 

However, other authors, such as Nakagawa et al. [48], reported that kynurenine induced endothelial dysfunction. Their results demonstrated that incubation with kynurenine (100 and 500 µM for 30 min and 10, 100, and 500 µM for 2 h) produced a decrease in acetylcholine vasodilatation and an increase in the production of O_2_^●−^ in rat aorta. These effects were ameliorated by ascorbic acid and the AhR antagonist CH-223191 but not by apocynin, a NADPH oxidase inhibitor. In another set of experiments using aorta rings without endothelium, the vasodilation induced by sodium nitroprusside was decreased, and O_2_^●−^ production was increased in the presence of kynurenine (100 µM) for 2 h. This dysfunction was reversed only with ascorbic acid and not with AhR antagonist or apocynin. Therefore, their results suggest that kynurenine-stimulated O_2_^●−^ production at the vascular level decreases both endothelium-dependent and independent vasodilation and that the AhR antagonist only reverts the effect in an aorta with an intact endothelium aorta. Therefore, the AhR pathway is involved in endothelial dysfunction due to oxidative stress induced by Kynurenine, whereas in the denuded aorta, Kynurenine induces oxidative stress not mediated by AhR.

2-(1’H-indole-3’-carbonyl)-thiazole-4-carboxylic acid methyl ester (ITE) is another AhR agonist involved in the regulation of immune responses and placental angiogenesis. Li et al. [43] showed that ITE inhibited the proliferation and viability of HUVECs and human artery endothelial cells (HUAECs). ITE also reduced migration in HUAECs. The authors postulated that this effect could be through AhR because CYP1A1 and CYP1B1, AhR target genes were upregulated. When AhR was silenced in vitro, ITE induced the same decrease in proliferation and viability in HUVECs, indicating that these effects were not mediated by AhR. However, AhR silencing was effective in inhibiting the ITE-induced decrease in proliferation and viability in HUAECs, suggesting these effects were produced by AhR activation. In conclusion, the authors proposed that ITE could regulate placental angiogenesis since ITE suppressed angiogenic responses of both types of cells, HUVECs, and HUAECs, in an AhR-independent and dependent pathway, respectively. 

On the other hand, Long et al. [44], in their search for new natural compounds to treat pulmonary arterial hypertension, showed that crude extract of *Tabernaemontana divaricata* vasodilated pulmonary arteries. When isolating its compounds, they found that 3′-Oxo-tabernaelegantine A (OTNA) produced selective vasodilation in the mouse pulmonary artery but not in the aorta. In fact, the concentration–response curve to OTNA produced vasodilatation in pulmonary arteries in an endothelium-dependent manner through activation of the PI3K/Akt/eNOS pathway. The incubation with the AhR agonist ITE (20 µM) blocked the relaxant effect of OTNA, and the AhR antagonist CH-223191 (10 µM) produced a synergistic effect with OTNA, indicating that the vasodilator mechanism of OTNA is partly by AhR inhibition. Furthermore, the incubation with ITE or AhR overexpression in human pulmonary arterial endothelial cells (HPAECs) and HUVECs inhibited the activation of PI3K/Akt/eNOS by OTNA. Their experiments also showed that OTNA is directly bound to AhR. Based on their results, the authors concluded that the AhR inhibition by OTNA could be a new therapeutic target to treat pulmonary arterial hypertension. 

6-Formylindolo[3,2-b]carbazole (FICZ). In the same context, Masaki et al. [46] studied the AhR pathway in pulmonary arterial hypertension (PAH). In their experiments in rats, subcutaneous administration of the endogenous AhR agonist FICZ (6-Formylindolo[3,2-b]carbazole) induced severe PAH only in a portion of the tested rats under normoxic conditions. FICZ, in combination with hypoxia, induced severe PAH in all of the tested rats, with an increase in right ventricular systolic pressure, medial wall thickness, and occlusive neointimal lesions. These changes were abolished in the AhR KO rats. Furthermore, the authors performed experiments in the SU5416/hypoxia (SuHx) rat model, which is widely used for reproducing many features of severe PAH, such as intimal and plexiform lesions. SU5416 is also a potent activator of AhR in vivo and in vitro, suggesting that the pathogenesis of PAH in the SuHx model may also involve activation of AhR, as well as inhibition of VEGFR2. Regarding ECs, AhR is highly expressed in ECs isolated from lungs of the SU5416/hypoxia rat model, suggesting that AhR activation in ECs may play an important role in the pathogenesis of PAH, affecting medial wall thickening, among other effects. To explore the involvement of AhR in the pathogenesis of PAH in humans, the authors performed an AhR luciferase reporter assay using the sera of patients with mild and severe PAH and compared it with healthy volunteers. AhR agonistic activity was significantly higher in patients with severe PAH than in those with mild PAH, and these were higher compared with healthy volunteers. Patients with higher AhR-Luc activity were significantly more susceptible to severe clinical events. The results from this complete paper suggest that AhR activation plays an essential role in the pathogenesis of PAH, and the AhR activity is directly proportional to the severity of the disease.

Indole-3-carbinol (I3C) is another AhR agonist found in cruciferous vegetables such as broccoli and has been attributed to an anti-obesity effect. In this sense, Wang et al. [50] studied the effect of I3C on mature adipocyte cultures, observing a decrease in the accumulation of triglycerides. This effect was associated with an increase in AhR and CYP1B1 expression. In addition, since the expansion of adipose tissue in obesity is accompanied by an increase in endothelial angiogenesis, the authors studied the effect of factors released by adipocytes on endothelial tube formation. For this purpose, adipocytes were treated with and without I3C (5–50 µM), and after 24 h, the adipocyte-conditioned medium was collected and used to culture endothelial cells. The endothelial tube formation was stained with calcein AM fluorescent dye and observed under the microscope. Their results indicated that adipocyte-conditioned medium favoured the formation of tube-like endothelial structures, confirming the regulation of endothelial function by substances released from mature adipocytes. However, when the adipocyte-conditioned medium was treated with I3C, less formation of tube-like endothelial structures was observed. Therefore, the authors concluded that I3C reduces the production of proangiogenic mediators by mature adipocytes, leading to a decrease in the formation of endothelial tube-like structures.

### 3.4. AhR Knockout Models

Biomedical research using knockout mice models offers valuable insight into elucidating the physiological function of a particular gene. In this section, studies using the AhR KO model were analysed to reveal the AhR function in the cardiovascular system, focusing on the endothelium. 

Agbor et al. [34] worked with ECAhR KO mice generated by crossing AhR floxed mice to mice expressing Cre recombinase driven by EC-specific promoter. They hypothesised that ECAhR KO mice would have a phenotype very similar to AhR KO since AhR is highly expressed in the endothelium. Their results indicated that ECAhR KO mice were hypotensive, and they ruled out that it was related to a decrease in sympathetic contribution or an increase in the nitric oxide (NO) pathway. The mechanism they proposed was reduced downstream signalling of angiotensin II. The authors reached this conclusion based on the results of their in vivo and ex vivo experiments. In the first case, acute injection of angiotensin II raised blood pressure in both ECAhR KO and control mice, with a lower increase in blood pressure after 30 min in the former group. They also chronically treated mice with ACE inhibitors, and both genotypes reduced their blood pressure. Once the treatment ceased, diastolic pressure remained lower in the ECAhR KO than in the control group. In their ex vivo experiments, the abdominal aorta from the ECAhR KO showed a lower vasocontractile response to angiotensin II (500 nM) compared to control. This result was only obtained in the aorta with perivascular adipose tissue. The authors explain these results because of the lower protein expression of angiotensin II receptor type 1 (ATR1) found in the abdominal aorta of ECAhR KO. However, it remains unexplained that angiotensin II has the same effect in both groups in the absence of perivascular adipose tissue. They speculate that loss of endothelial AhR may alter paracrine signalling and increase vasodilatory factors from adipose tissue. Although these authors do provide evidence that endothelial AhR plays a critical role in the response to angiotensin II and may, therefore, affect blood pressure control, the mechanism by which AhR regulates blood pressure remains unclear. 

Lund et al. [45] studied how altitude affects blood pressure in AhR KO mice. Their results indicated that AhR KO developed hypotension at a low altitude (225 m) and hypertension at a modest altitude (1632 m). At both altitudes, the AhR KO mice had high plasma endothelin-1 (ET-1) levels, which further increased at moderate altitudes. Their initial hypothesis was that AhR would attenuate the gene response to hypoxia, but this was not demonstrated by the results obtained. Their explanation is that increased ET-1 would lead to increased systemic hypertension in the absence of signs of pulmonary hypertension without polycythaemia and right ventricular hypertrophy. 

In addition, they studied the inactivation of AhR in HUVECs and lung microvessel ECs and found a decrease in preproET-1 mRNA levels, indicating that the increased plasma levels of ET-1 observed in AhR KO mice did not originate from pulmonary vessels. They speculate that the source may be hepatic, as the liver is also involved in ET-1 clearance, and the AhR KO mice have a smaller liver due to the persistent ductus venosus. Overall, the authors concluded that the AhR KO model is hypertensive when the mice were at modest altitudes and hypotensive at low altitudes.

Zhang et al. [52] worked in both a heterozygous and homozygous KO model and found hypotension in homozygous but not in heterozygous mice. They studied contraction to phenylephrine in the aorta, obtaining a greater response in the heterozygote. When NO synthesis was blocked, the increase in contraction to phenylephrine was greater in the AhR KO compared to WT. This effect was more potent in homozygotes. Consistently, the authors found higher expression of eNOS in the homozygote. To test whether the hypotension of the homozygote was caused by an increase in NO, the authors treated the mice with an eNOS inhibitor, but the increase in blood pressure was not sufficient to corroborate their hypothesis. So, they treated the mice with captopril (an angiotensin-converting enzyme (ACE) inhibitor), noting that the heterozygote lowered their blood pressure much more than the other group. In addition, the heterozygote had higher plasma renin and ACE activity compared to the other groups, which allowed the authors to conclude that renin–angiotensin system activation was the cause of the heterozygote not being hypotensive.

Moreover, AhR has been shown to regulate lipid metabolism and may, therefore, play a role in obesity. It is well known the association between endothelial dysfunction and obesity; however, little is known about the role of AhR in vascular alterations induced by obesity. A recent study using wild-type male mice fed a high-fat diet (HFD) for 10 weeks, conducted by Fernandes da Silva et al. [37], found an increase in AhR protein expression and a decreased endothelium-dependent vasodilation in the thoracic aorta. When AhR KO mice were fed an HFD, the authors observed that, in addition to being protected against dyslipidaemia, weight gain, and inflammation, the aorta of these mice did not show lower endothelium-dependent vasodilatation as was the case in the aorta of wild type male mice fed an HFD. Moreover, the aorta from Ahr KO mice had a lower contractile response to phenilephrine, which was reversed in the presence of an eNOS inhibitor, suggesting that there was increased NO bioavailability. To verify this, they measured both NO and ROS levels in the aorta by fluorescence and concluded that only the NO levels, but not ROS levels, were elevated in the AhR KO mice. The authors also observed that in HUVECs incubated with lysophosphatidylcholine (LPC), the main component of LDL and oxidized LDL decreased eNOS expression and NO production, which was abolished by the AhR antagonist, CH-223191 (1 µM). Furthermore, they found a reduced endothelium-dependent vasodilation in the aorta from WT incubated with LPC for 24 h. This effect was reversed in the presence of the AhR antagonist CH-223191 (1 µM). With all these results, they concluded that AhR deletion increased NO signalling, attenuating endothelial dysfunction induced by HFD.

Finally, Fehsel et al. [36], on the basis that the adverse effects of clozapine could be related to the activation of the AhR pathway, designed vascular reactivity experiments in wild-type and AhR KO mice aorta. They performed concentration-response curves to acetylcholine in the absence and presence of clozapine and observed a lower vasodilatation in the presence of clozapine, which even reduced the maximal effect (Emax) to acetylcholine. In AhR KO mice aorta, the presence of clozapine still reduced acetylcholine vasodilation without affecting Emax as in WT mice aorta. The authors attributed this difference to AhR activation. In WT mice aorta, acetylcholine curves were also performed in the presence of the AhR antagonist CH-223191, and a slight shift of the curve to the left was observed, indicating that AhR activity at the basal level opposes the vasodilation induced by acetylcholine. Aortic segments from WT mice were also incubated with clozapine and CH-223191, and although vasodilation to acetylcholine was improved, it was not comparable to control. The authors concluded that the identification of clozapine as an AhR agonist could explain the adverse effects of clozapine treatment; however, since clozapine is a muscarinic antagonist, part of the reduction in acetylcholine-induced vasodilation was due to this blockade and we consider that more experiments should be conducted to reach this conclusion. 

## 4. Conclusions

AhR activation seems to be responsible, at least in part, for endothelial dysfunction in patients with CKD since uremic toxins such as IS and IAA, as AhR agonists lead to increased oxidative stress and vascular inflammation. This detrimental effect of AhR activation on endothelial function has also been demonstrated in studies of pulmonary arterial hypertension. However, other AhR agonists, such as kynurenine, when it is endogenously released by cardiomyocytes and reaches the endothelium, are able to regenerate cardiac tissue. The cause of these mixed observations may be supported by the administration of higher concentrations of endogenous AhR agonists that do not reflect the plasma concentrations in healthy people. AhR KO models showed that AhR suppression can cause hypotension, hypertension, or attenuate HFD-induced endothelial dysfunction. Consequently, blocking the AhR pathway could improve the endothelial function in metabolic disorders such as obesity.

AhR is highly expressed in the endothelium, and the studies analysed in this review support a crucial role for it in the maintenance of vascular homeostasis, as well as that the endothelial responses to AhR activation can be different depending on the AhR agonist. This review provides new insight into AhR inhibition for the treatment of diseases with cardiovascular complications.

How the AhR pathway regulates vascular homeostasis requires further investigation, especially when endogenous ligands activate it at physiological concentrations.

## Figures and Tables

**Table 1:** List of selected manuscripts in alphabetical order.

Reference	Title	Sample/Model	AhR Agonist	AhR Antagonist	Main Results
(Agbor et al., 2011) [34]	Endothelial cell-specific aryl hydrocarbon receptor knockout mice exhibit hypotension mediated, in part, by an attenuated angiotensin II responsiveness	Abdominal aorta from WT and ECAhR KO mouse	---	---	Decrease in ATR1 protein expression in abdominal aorta from ECAhR KO. Hypotension in ECAhR KO unrelated to sympathetic or NO contribution. Reduced downstream signalling of Ang II in ECAhR KO.
(Dou et al., 2015) [35]	The cardiovascular effect of the uremic solute indole-3 acetic acid	HUVECs	IAA 5–50 μM	CH-223191 10 μM	Incubation with IAA increases COX-2 mRNA expression by AhR/p38MAPK/NFKB. IAA causes oxidative stress partially mediated by AhR activation. IAA is a predictor of cardiovascular mortality in patients with CKD.
(Fehsel et al., 2022) [36]	Activation of the aryl hydrocarbon receptor by clozapine induces preadipocyte differentiation and contributes to endothelial dysfunction	Aorta from AhR KO mouse	Clozapine 1 μM	CH-223191 10 μM	Decreased Emax in endothelial-dependent vasodilation in the presence of clozapine in WT but not in KO mouse. AhR activity opposes to endothelium- dependent vasodilation.
(da Silva et al., 2022) [37]	Aryl hydrocarbon receptor (AhR) activation contributes to high-fat diet-induced vascular dysfunction	Thoracic aorta from AhR KO mouse fed an HFD HUVECs	---	CH-223191 1 μM	Increase in AhR protein expression in thoracic aorta from WT mice fed an HFD. HFD decreases endothelial-dependent vasodilation in thoracic aorta from WT mice but not from AhR KO mice. Increase in NO production in thoracic aorta from AhR KO mice. In HUVECs, AhR antagonist reverts the decreased levels of NO induced by LPC. AhR KO protects against endothelial dysfunction induced by HFD.
(Gondouin et al., 2013) [38]	Indolic uremic solutes increase tissue factor production in endothelial cells by the aryl hydrocarbon receptor pathway	HUVECs	IS 1 mM IAA 50 μM	---	Increase in TF mRNA levels and eight genes regulated by AhR. IS and IAA increase TF via AhR proning to atherogenesis in CKD patients.
(Ito et al., 2016) [39]	Crucial Role of the Aryl Hydrocarbon Receptor (AhR) in Indoxyl Sulfate-Induced Vascular Inflammation	HUVECs ECAhR KO mouse	IS 0.2–2 mM	---	IS increases leukocyte–endothelial interactions through AP-1 transcriptional activity, and si AhR reverses it in HUVECs. Decrease in inflammation and leukocyte recruitment in ECAhR KO.
(Kim et al., 2017) [40]	Indoxyl sulfate (IS)-mediated immune dysfunction provokes endothelial damage in patients with end-stage renal disease (ESRD)	HUVECs	IS 1 mM	---	TNF-α produced by IS-conditioned monocytes from patients with CKD lead HUVECs to secrete CX3CL1 to further recruit immune cells, promote apoptosis and endothelial cell dysfunction.
(Koizumi et al., 2014) [41]	Aryl hydrocarbon receptor mediates indoxyl sulfate-induced cellular senescence in human umbilical vein endothelial cells	HUVECs	IS 500 μM	α-NF 10 μM CH-223191 10 μM	IS activates NADPH oxidase. IS decreases iNampt and Sirtuin 1 activity, which is reversed by AhR inhibition. AhR blockade may be useful for the treatment of cardiovascular complications in patients with CKD.
(Lano et al., 2020) [42]	Aryl Hydrocarbon Receptor Activation and Tissue Factor Induction by Fluid Shear Stress and Indoxyl Sulfate in Endothelial Cells	HUVECs	IS 200 μM Shear Stress	Genistein	IS and shear stress increase COX-2 and TF mRNA expression. IS induces TF activation with procoagulant effect. SS induces TF activation without procoagulant effect. IS and SS activate AhR by different mechanisms.
(Li et al., 2017) [43]	ITE Suppresses Angiogenic Responses in Human Artery and Vein Endothelial Cells: Differential Roles of AhR	HUVECs HUAECs	ITE 1 μM	---	ITE decreases the proliferation and viability of HUAECs in an AhR-independent manner and HUVECs in an AhR-dependent manner. ITE decreases AhR protein levels in HUAECs and HUVECs. ∙ ITE increases CYP1A1 and CYP1B1 mRNA levels in HUAECs and HUVECs.
(Long et al., 2021) [44]	3′-Oxo-tabernaelegantine A (OTNA) selectively relaxes pulmonary arteries by inhibiting AhR	Pulmonary artery and thoracic aorta from C57BL/6 mouseHUVECs & HPAECs	ITE 20 μM	OTNA CH-223191 10 μM	OTNA inhibits AhR and induces vasodilation in pulmonary artery by activating the PI3K/Akt/eNOS pathway. ITE blocks the vasodilation of OTNA, and CH-223191 produces a synergistic effect with OTNA. AhR inhibition could treat pulmonary arterial hypertension.
(Lund et al., 2008) [45]	Loss of the aryl hydrocarbon receptor induces hypoxemia, endothelin-1, and systemic hypertension at modest altitude	Plasma from AhR KO mouse HUVECs and microvascular ECs from lung	---	---	AhR KO at low altitude induces hypotension. AhR KO at modest altitude induces hypertension. Increase in ET-1 plasma levels in AhR KO does not originate in pulmonary vessels. Decreased in PreproET-1 mRNA levels in AhR-specific siRNA HUVECs and microvascular ECs from lung.
(Masaki et al., 2021) [46]	Aryl hydrocarbon receptor is essential for the pathogenesis of pulmonary arterial hypertension	ECs from SU5416/hypoxia AhR KO rat lung	FICZ 10 mg/kg/wk subcutaneous administration	---	ECs from lung of SU5416/hypoxia rat model have an increase in AhR expression. FICZ, in combination with hypoxia, induces severe PAH. AhR activity is directly proportional to the severity of pathogenesis of PAH.
(Nakagawa et al., 2021) [47]	Indoxyl sulfate induces ROS production via the aryl hydrocarbon receptor-NADPH oxidase pathway and inactivates NO in vascular tissues.	Thoracic aorta from rat	IS 250–1000 μM	CH-223191 10 μM	Increase in O_2_^●−^ production by IS through activation of NADPH oxidase. Decreased endothelium-dependent vasodilation in the presence of IS that was partially reversed by AhR antagonist.
(Nakagawa et al., 2022) [48]	Acute Kynurenine Exposure of Rat Thoracic Aorta Induces Vascular Dysfunction via Superoxide Anion Production	Thoracic aorta from rat	Kynurenine 10–500 µM	CH-223191 10 μM	Kynurenine increases O_2_^●−^ production, leading to decreased endothelium-dependent vasodilation.
(Nguyen et al., 2022) [49]	Aryl Hydrocarbon Receptor Inhibition Restores Indoxyl Sulfate-Mediated Endothelial Dysfunction in Rat Aortic Rings.	Aorta from rat	IS 10–300 μM	CH-223191 10 μM	Increase in O_2_^●−^ production by IS through activation of NADPH oxidase. Decreased endothelium-dependent vasodilation only at IS 300 μM.
(Wang et al., 2016) [50]	Suppression of Lipid Accumulation by Indole-3-Carbinol Is Associated with Increased Expression of the Aryl Hydrocarbon Receptor and CYP1B1 Proteins in Adipocytes and with Decreased Adipocyte-Stimulated Endothelial Tube Formation	Murine preadipocyte cell line 3T3-L1 Human endothelium-derived cell line EA hy926	I3C 50 μM	---	Decrease in pro-angiogenic mediators in adipocytes incubated with I3C. Decrease in endothelial angiogenesis when ECs are incubated with I3C adipocyte conditioned medium.
(Zhang et al., 2022) [51]	Kynurenine promotes neonatal heart regeneration by stimulating cardiomyocyte proliferation and cardiac angiogenesis	CMECs from mouse AhR KO mouse	Kynurenine from cardiomyocytes	---	Kynurenina transport between cardiomyocytes and EC. ∙ Increase in VEGFA gene expression by AhR activation in EC. Decrease in VEGFA in cardiac endothelium from AhR KO.
(Zhang et al., 2010) [52]	An activated renin–angiotensin system maintains normal blood pressure in aryl hydrocarbon receptor heterozygous mice but not in null mice	Thoracic aorta from AhR^+/+^, AhR^+/−^ and AhR^−/−^ mouse	---	---	Increase in eNOS protein expression in AhR^−/−^mouse. ∙ Further decrease in mean arterial pressure in AhR^−/−^ than AhR^+/−^ mouse. Further increase in response to ACE inhibitor or ETA antagonist of AhR^+/−^ than AhR^−/−^ mouse.

WT: wild type; ECAhR: Endothelial cell-specific AhR knockout; ATR1: Angiotensin II receptor type 1; NO: Nitric oxide; Ang II: Angiotensin II; HUVECs: Human Umbilical Vein Endothelial Cells; -; Emax: maximal effect; CKD: Chronic kidney disease; HFD: High-fat diet; LPC: Lysophosphatidylcholine; ECs: Endothelial cells; IAA: Indole-3-acetic acid; TF: tissue factor, IS: Indoxyl sulfate, α-NF: α-naphthoflavone; (iNampt): intracellular nicotinamide phosphoribosyltransferase; HUAECs: Human artery endothelial cells; HPAECs: Human pulmonary artery endothelial cells; CMECs: Cardiac microvascular endothelial cells; ITE: 2-(1′H-indole-3′-carbonyl)-thiazole-4-carboxylic acid methyl ester; FICZ: formylindolo[3,2-b]carbazole; I3C: Indole-3-carbinol.

## Data Availability

No new data were created.

## Abbreviations

AhR	Aryl hydrocarbon receptor
ACE	Angiotensin-converting enzyme
AhRR	AhR repressor
AP-1	Activator protein-1
ARNT	Aryl Hydrocarbon Receptor Nuclear Translocator
ATR1	Angiotensin II receptor type 1
CKD	Chronic kidney disease
CMECs	Cardiac microvascular endothelial cells
CMs	Cardiomyocytes
CVDs	Cardiovascular diseases
ECs	Endothelial cells
eNOS	Endothelial nitric oxide synthase
ET-1	Endothelin-1
FICZ	Formylindolo[3,2-b]carbazole
HFD	High-fat diet
HPAECs	Human pulmonary artery endothelial cells
Hsp90	Heat shock protein 90
HUAECs	Human artery endothelial cells
HUVECs	Human umbilical vein endothelial cells
I3C	Indole-3-carbinol
IAA	Indole-3-acetic acid
IS	Indoxyl sulfate
ITE	2-(1′H-indole-3′-carbonyl)-thiazole-4-carboxylic acid methyl ester
NO	Nitric oxide
O_2_^●−^	Superoxide anion
OTNA	3′-Oxo-tabernaelegantine A
TCDD	2,3,7,8-tetrachlorodibenzo-*p*-dioxin
TF	Tissue factor
VEGFA	Vascular endothelial growth factor A
XRE	Xenobiotic response element
α-NF	α-naphthoflavone
